# The role of routine cranial CT imaging in geriatric head trauma: a retrospective analysis from a level I trauma centre in Germany

**DOI:** 10.1016/j.bas.2025.105606

**Published:** 2025-09-12

**Authors:** Josina Straub, Julia Elisabeth Lenz, Leopold Henssler, Kristina Gerhardinger, Lisa Klute, Borys Frankewycz, Amr Mohamed, Volker Alt, Daniel Popp, Siegmund Lang, Maximilian Kerschbaum

**Affiliations:** a Clinic and Policlinic for Trauma Surgery, University Hospital Regensburg, Germany; b Department of Clinical Acute and Emergency Medicine, University Hospital Regensburg, Germany

**Keywords:** Geriatric patients, Intracranial bleeding, Head trauma, Anticoagulation, Risk factors, Number needed to screen

## Abstract

**Introduction:**

Geriatric patients with head trauma have an increased risk of intracranial injuries. Due to impaired clinical evaluability and the widespread antithrombotic use, cranial computertomography (cCT) is frequently performed.

**Research question:**

This study aims to determine the prevalence and risk factors for intracranial haemorrhage and to calculate the number needed to screen (NNS) for its detection in a geriatric cohort.

**Methods:**

A retrospective analysis was conducted on patients aged ≥65 years presenting to a level I trauma centre in Germany between January 2020 and October 2024 after a head impact and undergoing cCT imaging.

**Results:**

Among 2474 patients, 62.9 % were aged ≥80 years and 56.1 % were female. Intracranial haemorrhage occurred in 9.3 %, with 1.5 % requiring surgery. Risk factors for intracranial haemorrhage included female gender (OR = 1.4; p = 0.014), impaired consciousness (OR = 3.5; p < 0.001), presentation via resuscitation room (OR = 6.5; p < 0.001) and cervical spine injury (OR = 2.2; p < 0.001). The NNS for detecting intracranial haemorrhage was 10.7 in patients aged ≥65 years and 11.4 in those aged ≥80 years. The NNS in patients without impaired consciousness was 19.2 for those aged ≥65 years and 20.5 for those aged ≥80 years. The NNS for surgical intervention was 65.1 in patients aged ≥65 years and 111.1 in those aged ≥80 years.

**Discussion and conclusion:**

Intracranial haemorrhage is a common consequence of head trauma in older patients. Despite its high frequency, routine cCT is crucial for timely identification of acute intracranial pathologies, with the low NNS highlighting its diagnostic value and justifying its widespread use to optimize patients’ outcomes.

## Highlights

• cCT imaging is crucial for timely identification of acute intracranial pathologies.
• cCT imaging is directly influences clinical decision-making.
• Risk factors: female sex, impaired vigilance and cervical spine injury.
• Low NNS underlines the need of routine cCT imaging in geriatrics with head impact.

## 1 Introduction

According to an estimate by the United Nations Department of Economic and Social Affairs, 20 % of the global population will be older than 65 years by 2050 (Shi et al., 2023). Trauma is a frequent reason for emergency department visits and hospital admissions in the geriatric population (Shi et al., 2023). Therefore, falls in older adults remain a significant public health issue and represent the leading cause of injury within this demographic (Prabhakaran et al., 2020). Approximately one-third of all patients aged 65 years and older experience at least one fall per year, with half of these individuals presenting to an emergency department for treatment (de Wit et al., 2020). In addition to fall-related fractures, intracranial haemorrhage is a common injury patterns in older patients, usually caused by minor trauma mechanisms such as ground-level falls (Schindler et al., 2023). The geriatric population is less likely to protect the head with their arms during a fall (Hruska and Ruge, 2018). Furthermore, cerebral atrophy leads to increased tension on the bridging veins, which are more vulnerable in older adults compared to younger individuals (Adhiyaman et al., 2002). However, a significant number of patients are unable to recall whether they sustained a head impact, owing to factors such as memory deficits, cognitive deterioration and potential loss of consciousness (Turchiaro et al., 2023). The Canadian Computertomography Head Rule (CCHR), published in 2001, was designed to identify patients with head injuries who could be safely cleared without brain imaging (Stiell et al., 2001). Inclusion criteria for cranial CT imaging (cCT imaging) are loss of consciousness, definite amnesia, witnessed disorientation and an initial Glasgow Coma Scale (GCS) score < 15 (Stiell et al., 2001). Following the publication of CCHR, the number of cCT scans during emergency department visits for injury-related conditions increased threefold with no significant rise in the detection of emergent conditions (Korley et al., 2010). While the Canadian CT Head Rule (CCHR) is designed for and useful in a patient cohort younger than 65 years, it cannot exclude the need for cranial CT in geriatric patients with amnesia related to the event and potentially concomitant dementia. (Turchiaro et al., 2023). Moreover, the Scandinavian guidelines for the initial management of minimal, mild, and moderate head injury classify patients according to Glasgow Coma Scale (GCS) scores and the presence of clinical risk factors. They aim to safely reduce unnecessary cCT scans by stratifying patients based on factors such as age ≥65, anticoagulant use, loss of consciousness, repeated vomiting, or signs of skull fracture. Patients with minimal head injury (GCS 15 without risk factors) may be discharged without imaging, while those with mild or moderate injury and additional risk factors require CT and possibly hospital observation (Undén et al., 2013). Older patients are often treated with antithrombotic medications due to vascular or cardiac diseases, primarily for the prevention and treatment of thromboembolic events (Schindler et al., 2023). The use of antiplatelet agents for primary prevention of cardiovascular diseases is particularly widespread in Germany (McNeil et al., 2018). Approximately 50 % of individuals aged 70 or older take antiplatelet medications daily (McNeil et al., 2018). Antithrombotic medication interfere with normal blood coagulation, thereby markedly increasing the risk of bleeding after traumatic events (Schindler et al., 2023). Underestimating the severity of a intracranial bleeding in patients receiving anticoagulation therapy is particularly dangerous, as impaired coagulation can lead to a significant progression of an intracranial bleeding (de Wit et al., 2020). In the presence of neurological symptoms and risk factors, non-contrast cCT is considered the gold standard in the primary diagnosis of intracranial bleeding for the assessment of intracranial injuries (Wiegele et al., 2019). However, the management of older patients, particularly those without risk factors and neurological symptoms or pre-existing dementia can be further challenging (Higgs and Gilleard, 2017). This creates challenges for physicians, as standard practice is to perform a CT scan even when there is no clear evidence of trauma, leading to unnecessary and costly imaging.

Therefore, this study aimed to investigate the prevalence of intracranial bleeding as well as its associated risk factors in older patients after a trauma with non-excludable subsequent head trauma. Moreover, the number needed to screen (NNS) for the detection of intracranial bleeding, for detection of intracranial bleeding in asymptomatic patients as well as for patients requiring surgical intervention was calculated. A subgroup analysis was performed for patients aged ≥65 years and ≥80 years. By surveying this geriatric group, we aimed to gain insights into the individual factors and the NNS for intracranial bleeding. Understanding these factors is essential for the accurate diagnosis of older patients presenting to the emergency department following head trauma, enabling the implementation of targeted interventions to optimize patient care outcomes.

## 2 Methods

### 2.1 Data collection

This retrospective analysis examined all older patients aged 65 years or older presenting to our emergency department of a level I trauma centre following a trauma with subsequent head impact and undergoing cCT imaging between January 2020 and October 2024. Patients were selected based on clinical judgment and local imaging protocols. A complete registry of patients with head trauma who did not receive CT was not available due to the retrospective nature of data collection.

Data collection encompassed demographic characteristics, antithrombotic therapy administered to the older patients as well as level of consciousness, pupillary response and sensomotoric function. In this study, anisocoria and a delayed pupillary reaction were classified as pathological pupillary responses. Additionally, a reduction in consciousness was assessed using the GCS, with a GCS score of ≤13 defined as impaired consciousness. Sensory or motor deficits were defined as any sensory or motor impairment documented in the admission records. In the following, asymptomatic patients were defined as individuals presenting with a GCS score ≥ 14, exhibiting normal and bilaterally symmetrical pupillary light reflexes and reporting no pain.

Patients presenting with head trauma were evaluated for cCT imaging. Imaging was systematically performed in individuals with simultaneous long-term anticoagulation therapy and pre-existing severe dementia, where clinical neurological assessment was deemed unreliable, as well as in those demonstrating impaired consciousness or pathological pupillary findings. This protocol was implemented to ensure thorough evaluation of patients at increased risk or with compromised clinical assessment. In cases of cervical spine pain or patients with inadequate assessment due to reduced consciousness or pre-existing dementia, both cCT and cervical spine CT were performed. In patients with painful midface and suspected midface injuries, imaging was extended to include a midface CT. For patients presenting via resuscitation room, a polytrauma CT scan was routinely conducted.

The presence of intracranial haemorrhage was assessed, along with its clinical management and outcomes, including surgical intervention, in-patient monitoring or discharge. Additionally, the occurrence of concurrent cervical spine injuries was systematically documented. The frequency of intracranial bleeding, its associated risk factors as well as the NNS for the detection of bleeding, detection of bleeding in asymptomatic patients and for the surgical management of intracranial haemorrhage were recorded.

### 2.2 Statistical analysis

The data were analysed to assess the prevalence of intracranial bleeding following a fall with subsequent head impact in patients aged ≥ 65 years and identify associated risk factors in older patients. Furthermore, the analysis included the evaluation of NNS for detecting intracranial bleeding and the need for surgical treatment, as well as NNS for detecting intracranial bleeding in patients without antithrombotic drug use and in patients with normal consciousness levels and pupillary response after head trauma. Age-specific analyses were performed for patients aged ≥ 65 years and ≥ 80 years to explore potential differences between these groups.

Statistical analyses were performed using SPSS (IBM SPSS Statistics, version 30.0.0, Zurich, Switzerland). Categorical variables were presented as frequencies and percentages, while descriptive statistics were used to summarize the characteristics of intracranial bleeding in older patients following head trauma and cCT imaging. Continuous variables were reported as means with standard deviations and categorical variables as counts and percentages. The odds ratio (OR) was utilized to assess risk factors associated with intracranial bleeding after head trauma in older patients. The significance level was defined at 5 %.

## 3 Ethical approval

The study was conducted in accordance with the ethical standards of the Declaration of Helsinki. A positive ethical approval has been obtained from the local ethics committee of the University of Regensburg (24-4010-104).

## 4 Results

A total of 2474 older patients with head trauma underwent cCT in the emergency department. The mean age was 81.4 years and 56.1 % were female. Pre-existing dementia was documented in 19.2 %. Overall, 55.0 % of patients received long-term antithrombotic therapy, of whom 27.3 % were treated with antiplatelet agents, 27.1 % with oral anticoagulants, and 0.8 % with a combination of both. Overall, 60.8 % presented to the emergency department following a trip-and-fall, while 16.6 % were admitted via the resuscitation room. Treatment at intensive care unit (ICU) was required in 6.9 % and 1.9 % died during their hospital stay. The detailed patient-specific characteristics are presented in Table 1.

**Table 1:** Patient-specific characteristics.

characteristics	N or average	% or SD
age	81.4	7.9

female	1388	56.1

pre-existing dementia	474	19.2

consumption of alcohol	109	4.4

anticoagulation	1360	55.0

*type of anticoagulation*		
none	1109	44.8
antiplatelet agents	675	27.3
oral anticoagulants	671	27.1
both	19	0.8

*mechanism of injury*		
tripping fall	1503	60.8
nursing home fall	568	23.0
bicycle fall	84	3.4
traffic accident	319	12.9

presentation via resuscitation room	411	16.6

intensive care unit monitoring	171	6.9

death	46	1.9

Following head trauma, 20.9 % of the patients presented with impaired consciousness upon arrival at the emergency department and 5.2 % demonstrated an abnormal pupillary reaction. Additionally, 10.2 % of cases reported cervical spine pain and 1.5 % exhibited a sensomotoric deficit. All patients underwent cCT imaging, with 68.0 % having received both cCT and cervical spine CT imaging. The detailed diagnostic-specific parameters are presented in Table 2.

**Table 2:** Diagnostic-specific parameters.

characteristic	N	%
impaired consciousness	517	20.9

*pupil reaction*		
normal	2347	94.9
anisocoria	93	3.8
delayed pupil reaction	34	1.4

cervical spine pain	253	10.2

sensomotoric deficit	38	1.5

*CT-imaging*		
cCT	361	14.6
cCT & cervical CT	1251	50.5
cCT, cervical spine & midface CT	432	17.5
polytrauma-CT	430	17.4

Overall, intracranial bleeding was identified in 9.3 % of cases, with subarachnoid haemorrhage being the most common with 3.1 %. Additionally, 46.1 % of patients presented with soft tissue facial injuries. Among patients with intracranial haemorrhage, 16.5 % received neurosurgical treatment and 70.6 % required monitoring (Table 3).

**Table 3:** Patterns of injury.

characteristics	N	%
intracranial bleeding	231	9.3

*type of intracranial bleeding*		
none	2243	90.7
intracerebral haemorrhage	39	1.6
subarachnoid haemorrhage	77	3.1
epidural hematoma	8	0.3
subdural hematoma	44	1.8
combination	63	2.5

*associated injuries*		
none	816	33.0
soft tissue facial injuries	1140	46.1
facial skull fractures	399	16.1
Soft tissue injuries and facial skull fractures	119	4.8
cervical spine injury	112	5.2

*Treatment intracranial bleeding*		
operation	38	16.5
monitoring	163	70.6
discharge	30	12.9

Female gender (OR = 1.4; p = 0.014), impaired consciousness (OR = 3.5; p < 0.001), presentation via resuscitation room (OR = 6.5; p < 0.001) and concomitant cervical spine injury (OR = 2.2; p < 0.001) were identified as risk factors for intracranial bleeding after a head impact in older patients. Long-term anticoagulation therapy (OR = 0.9; p = 0.68), age ≥ 80 years (OR = 1.1; p = 0.07) as well as pre-existing dementia (OR = 0.8; p = 0.36) and sensomotoric deficits (OR = 1.1; p = 0.80) could not be identified as risk factors (Table 4).

**Table 4:** Risk factors for intracranial bleeding in older patients after head trauma.

characteristics	OR	p-value	95 % CI
female	1.4	0.014	1.1–1.8
age ≥ 80 years	1.1	0.07	0.9–1.2
anticoagulation	0.9	0.68	0.7–1.2
impaired consciousness	3.5	<0.001	2.6–4.6
pre-existing dementia	0.8	0.36	0.6–1.2
presentation via resuscitation room	6.5	<0.001	4.9–8.7
sensomotoric deficit	1.1	0.80	0.4–3.3
injury cervical spine	2.2	<0.001	1.4–3.5

In patients aged ≥65 years, the NNS for cCT imaging to detect an intracranial haemorrhage was 10.7, the NNS for detecting an intracranial haemorrhage in asymptomatic patients was 19.2, the NNS for intracranial haemorrhage without antithrombotic drug use occurred in 20.8 and the NNS for identifying cases requiring surgical intervention due to intracranial haemorrhage was 65.1.

In patients aged ≥80 years, the NNS for cCT imaging to detect an intracranial haemorrhage was 11.4, the NNS for detecting an intracranial haemorrhage in asymptomatic patients was 20.5, the NSS intracranial bleeding without antithrombotic drug use occurred in 19.8 and the NNS for identifying cases requiring neurosurgical treatment due to intracranial haemorrhage was 111.1. (Table 5).

**Table 5:** Number needed to screen (NNS) of cranial computertomography for detection of intracranial bleeding, detection of intracranial bleeding in asymptomatic patients and patients without antithrombotic drug use as well as operative treatment for intracranial bleeding in patients ≥65 and ≥ 80 years.

characteristics	NNS
*patients ≥65 years*	
intracranial bleeding	10.7
intracranial bleeding without impaired consciousness	19.2
intracranial bleeding without antithrombotic drugs	20.8
operative treatment for intracranial bleeding	65.1

*patients ≥80 years*	
intracranial bleeding	11.4
intracranial bleeding without impaired consciousness	20.5
intracranial bleeding without antithrombotic drugs	19.8
operative treatment for intracranial bleeding	111.1

## 5 Discussion

In this study of 2474 older patients undergoing cCT imaging due to head impact, we identified a substantial burden on emergency departments and hospitals caused by intracranial bleeding. Several risk factors for the presence of intracranial haemorrhage were identified, with the most pronounced being female sex, impaired consciousness, presentation via the resuscitation room and concomitant cervical spine injury. The low NNS highlights the necessity of routine cCT imaging in older patients with head impact, even after minor trauma.

### 5.1 Anthropometric data and diagnostic characteristics

Life expectancy is rising, resulting in a higher incidence of accidents among the geriatric population (Schindler et al., 2023). Falls continue to be a significant public health issue and are the leading cause of injury in older adults (Prabhakaran et al., 2020). Around one-third of individuals aged 65 and older experience at least one fall annually, with half of them seeking treatment at an emergency department (de Wit et al., 2020). In addition to fall-related fractures, intracranial haemorrhage is a prevalent injury pattern in older patients, usually caused by minor trauma (Schindler et al., 2023). In our study of 2474 older patients undergoing cCT imaging after head trauma, the average age was 81.4 years, with 56 % being female. This is comparable to literature (Schindler et al., 2020, 2023). Schindler et al. also described a similar prevalence between men and women affected following geriatric falls with head impact (Schindler et al., 2020). Additionally, 19.2 % had a pre-existing diagnosis of dementia, while 55.0 % were receiving long-term anticoagulation therapy, with a balanced distribution between antiplatelet agents and oral anticoagulants. The literature reports similar rates of dementia in elderly individuals, with approximately 20 % affected (GIOFFRÈ et al., 2018). The high prevalence of chronic dementia in the elderly may contribute to delays in both presentation of symptoms and treatment (Higgs and Gilleard, 2017). Therefore, obtaining an accurate medical history and performing a comprehensive physical examination in elderly patients with dementia can present significant challenges (Yee and Jain, 2025). The Scandinavian guidelines for the initial management of minor, mild, and moderate traumatic brain injury recommend considering a 12-h clinical observation period as an alternative to immediate cranial CT imaging in selected older adults not receiving antithrombotic therapy (Undén et al., 2013). Given the retrospective design of the study, structured observation periods could not be reliably identified or quantified, precluding a direct assessment of observation as an alternative to imaging in this subgroup. Consequently, the current data do not allow firm conclusions regarding the safety or effectiveness of such an approach. Nonetheless, retrospective as well as prospective studies have validated the Scandinavian guidelines and confirmed their clinical utility in adults (Undén et al., 2013; Minkkinen et al., 2019). The literature indicates lower rates of anticoagulation, with approximately 27.5 % of patients using anticoagulants and 37.4 % using antiplatelets (Turchiaro et al., 2023). In our study, a mortality rate of 1.9 % was observed, with 6.9 % of patients subsequently requiring intensive medical care at the ICU. While literature reports a comparable mortality rate with 2.4 %, slightly higher rates of ICU admissions with 8.4 % were observed (Eryurt et al., 2023; Lee et al., 2022). Additionally, 5.2 % demonstrated a concomitant cervical spine injury resulting from the fall. However, Kerschbaum et al. demonstrated in a nationwide analysis about geriatric falls a higher rate of cervical spine injury with 18.6 % (Kerschbaum et al., 2024). The increased rate of cervical spine injuries may be attributed to the differences between a nationwide study and a monocentric study conducted at a level I trauma centre.

Among the mechanism of accident, trip-related falls were the most prevalent, accounting for 60.8 %, while 12.9 % sustained head impact due to a traffic accident. Yee et al. also reported ground-level falls as the most common cause of falls, with traffic accidents as the second most frequent (Yee and Jain, 2025). In contrast, our analysis identified falls in nursing homes, classified as low-energy trauma, as the second leading cause, accounting for 23 %. Brucoli et al. described, in their retrospective analysis of facial fractures, a ground-level fall as mechanism of injury most frequently, with zygomatic fractures occurring most frequently at 30 % (Brucoli et al., 2020). In our analysis, concomitant fractures of the facial skull were observed in 16.1 % of cases.

The observed intracranial haemorrhage rate of 9.3 % is consistent with prior studies and supports the continued use of cCT in older adults with head trauma in selected clinical settings (Turchiaro et al., 2023; Eryurt et al., 2023). However, the lack of data on non-imaged patients limits broader conclusions about imaging thresholds or population-level incidence. Contrary to existing literature, our study identified subarachnoid haemorrhage as the most prevalent type of intracranial bleeding, accounting for 3.1 %, followed by a combination of multiple bleeding types with 2.5 %. In the literature, however, subdural haemorrhage is described as the most frequent bleeding type in older patients, occurring in approximately 6 % of a fall with subsequent head impact (Eryurt et al., 2023; Kirkpatrick and Pearson, 1978). Furthermore, advancing age results in progressive brain atrophy, which creates more space for increased bleeding, potentially causing elderly patients to seek medical attention later (Yee and Jain, 2025). This delay in presentation, combined with subsequent delays in initiating treatment, contributes to the higher morbidity and mortality observed in geriatric population (Kirkpatrick and Pearson, 1978). In our study, a neurosurgical indication for surgery was recorded in 16.5 %, significantly higher than the 1.5 % observed in the prospective cohort study by Turchiaro et al. (2023). This observation may be influenced by the fact that some older patients, following a fall with simultaneous head impact and subsequent intracranial haemorrhage, are transferred to our emergency department for further management by the neurosurgery department.

### 5.2 Risk factors

The analysis of risk factors for intracranial haemorrhage following falls with head impact in older patients is a crucial step for improving subsequent diagnosis, treatment and prevention of such events (Mak et al., 2012). In our study, female gender (OR = 1.4; p = 0.014), impaired consciousness (OR = 3.5; p < 0.001), presentation via resuscitation room (OR = 6.5; p < 0.001) and concomitant cervical spine injury (OR = 2.2; p < 0.001) were identified as risk factors for intracranial bleeding after head trauma in older patients. Comparable to our analysis, Turchiaro et al. identified a diminished GCS (OR = 3.2, p < 0.001) as an significant risk factor for intracranial haemorrhage (Turchiaro et al., 2023). In contrast to our analysis, where a GCS ≤ 13 was defined as impaired consciousness, a GCS < 15 was used to define reduced consciousness in the study of Turchiaro et al. (2023). Regardless, in both cases, reduced consciousness was identified as risk factor for intracranial haemorrhage following a head impact in older patients. In contrast to our analysis, where long-term anticoagulation therapy (OR = 0.9; p = 0.68) was not identified as a risk factor, Turchiaro et al. also recognized the antiplatelet use (OR = 1.8; p < 0.001), both anticoagulant and antiplatelet use (OR = 2.4; p < 0.001) as well as alcohol consumption (OR = 1,6; p < 0.001) as risk factors for intracranial haemorrhage (Turchiaro et al., 2023). In contrast to our analysis, male gender (OR = 1,6; p < 0.001) was identified as a risk factor in literature, whereas our analysis recorded female gender (OR = 1.4; p = 0.014) as risk factor for intracranial haemorrhage in older patients after head impact (Turchiaro et al., 2023; Hwang et al., 2015). The cause for various gender as risk factor remains unclear, although this may be attributed to the differences between the countries. The absence of pre-existing dementia as a risk factor in both our analysis (OR = 0.8; p = 0.36) and in the literature (OR = 0.9; p = 0.55) suggests dementia may complicate clinical assessment and evaluation (Turchiaro et al., 2023; Higgs and Gilleard, 2017). However, it does not appear to directly contribute to the occurrence of intracranial haemorrhage. Additional studies further established the use of antidepressants (HR = 1.35) as a risk factor for traumatic intracranial haemorrhage in geriatric population (Taipale et al., 2017).

To the best of our knowledge, our study is the first to investigate the NNS for performing a cCT scan to detect and identify the need for treatment of intracranial haemorrhage by neurosurgical operation. In addition to a subanalysis of two different age groups, a subanalysis of neurologically asymptomatic older patients was conducted. A NNS of 10.7 for patients aged ≥65 y ears and 11.4 for patients aged ≥80 years for cCT imaging to detect intracranial haemorrhage was identified. The NNS for detecting intracranial haemorrhage in asymptomatic patients was 19.2 for patients aged ≥65 years and 20.5 for patients aged ≥80 years. Among patients without antithrombotic medication, the NNS to identify one case of intracranial haemorrhage was 20.8 in individuals aged ≥65 years and 19.8 in those aged ≥80 years. The NNS for identifying cases requiring surgical intervention due to intracranial haemorrhage was 65.1 for patients aged ≥65 years and 111.1 for patients aged ≥80 years.

This underlines the essential role of cCT imaging in older patients with head trauma, including those who are neurologically asymptomatic. Consequently, timely cCT imaging should be implemented in all emergency departments for older patients with head impact, even following low-energy trauma, to enhance diagnostic accuracy, guide treatment decisions and potentially prevent delayed interventions.

### 5.3 Limitations

Despite multiple advantages, this study has several limitations. As a retrospective analysis, it is subject to potential selection bias and limitations in data availability. The study population is limited to individuals who underwent cCT and no systematic record of patients managed without imaging was available. As such, the proportion of all older patients with head trauma presenting to the emergency department during the study period could not be determined. The exclusion of asymptomatic patients without antithrombotic therapy, which may influence the applicability of our findings related to antithrombotic treatment as a risk factor is one limitation. Another limitation was the failure to conduct long-term evaluations of post-discharge clinical outcomes. Additionally, being a single-centre study conducted in the emergency department of a level I trauma centre in Germany, the findings may not be generalizable to other healthcare settings. To enhance external validity and confirm these results, a multicentre study is warranted.

## 6 Conclusion

cCT imaging constitutes a key component in the diagnostic evaluation of older adults with head trauma, especially in individuals with impaired clinical assessability, cognitive dysfunction, or atypical presentation. While we do not propose a universal imaging mandate, the data support maintaining a low threshold for cCT use in selected patients. Despite the high frequency of cCT imaging, its consistent use remains essential, as it enables the timely identification of acute intracranial pathologies and directly influences clinical decision-making in selected patients. The relatively low NNS observed in this study underscores the diagnostic value of routine cCT in appropriately triaged patients, justifying its implementation to prevent potential complications and optimize patient outcomes. This study highlights the need for prospective, risk-stratified research to further optimize imaging strategies in this growing patient population.

## Informed consent

Not necessary.

## Ethics approval

Ethical Approval from the University Regensburg (24-4010-104).

## Data availability statement

Available on request.

## Authors’ contribution

The manuscript was created by JS, SL and MK. JS, AM, SL and MK performed the statistical analysis and designed the study. JS, SL, KG, LK, LH, JL, VA, BF, DP and MK conceived of the study, helped to draft the manuscript and participated in its design. All authors read and approved the final manuscript.

## Statements and declarations

No funding was received for conducting this study. The authors have no relevant financial or non-financial interests to disclose.

## Funding

No funding was received for conducting this study.

## Declaration of competing interest

The authors declare that they have no known competing financial interests or personal relationships that could have appeared to influence the work reported in this paper.
